# Perceived neighborhood disorder and achieving HIV viral suppression among adults living with HIV: A cross-sectional study

**DOI:** 10.1371/journal.pgph.0004060

**Published:** 2024-12-19

**Authors:** Linda Jepkoech Kimaru, ChengCheng Hu, Sudha Nagalingam, Priscilla Magrath, Elizabeth Connick, Kacey Ernst, John Ehiri

**Affiliations:** 1 Department of Health Promotion Sciences, The University of Arizona, Tucson, Arizona, United States of America; 2 Department of Epidemiology and Biostatistics, The University of Arizona, Tucson, Arizona, United States of America; 3 El Rio Special Immunology Associates, El Rio Health, Tucson, Arizona, United States of America; 4 Department of Medicine, The University of Arizona, Tucson, Arizona, United States of America; PLOS: Public Library of Science, UNITED STATES OF AMERICA

## Abstract

Adherence to antiretroviral therapy (ART) is crucial for achieving and maintaining viral suppression in people living with HIV (PLWH). While individual factors affecting HIV viral suppression have been extensively studied, there is less attention on community-level factors, specifically perceived neighborhood disorder. This study aims to assess the relationship between perceived neighborhood disorder and achieving virologic suppression among people living with HIV. One hundred and eighty-eight PLWH 18 years of age and older from two HIV clinics completed a cross-sectional study. We assessed perceptions of neighborhood disorder, ART self-efficacy, social support, alcohol and drug use, depression, HIV stigma, provider-patient relationship, demographics, and length at the zip code. HIV viral loads were obtained from the clinical record. The analysis involved the use of Fisher’s Exact test, Spearman’s Rank test, Wilcoxon rank sum test, and Firth logistic regression. All analyses were conducted using STATA 17. Most participants were male (79%), white (62%), and identified as non-Hispanic (66%). Individuals with no perceived neighborhood disorder had median scores of 10 for integration and perseverance in ART self-efficacy. Those with high perceived disorder displayed decreased scores of 8.4 and 8.3 for integration and perseverance respectively. Both integration and perseverance showed statistically significant negative correlations with perceived neighborhood disorder, (Spearman’s rho -0.2966; p<0.000 and -0.2387; p = 0.0010 respectively). Individuals with virologic suppression (n = 167) reported significantly lower perceived neighborhood disorder scores (median = 0.9 [IQR: 0.2–2.0]) compared to those without virologic suppression (n = 10, median = 3.2 [IQR: 2.4–4], p = 0.0012). The study highlights a notable correlation between perceived neighborhood disorder, ART adherence self-efficacy, and virologic suppression. This indicates that improving HIV treatment outcomes needs to extend beyond individual-level factors and include strategies to address neighborhood-level conditions. Public health policies and programs should consider the broader social and environmental contexts in which people living with HIV reside.

## Introduction

The management of Human Immunodeficiency Virus (HIV) remains a global health challenge, with antiretroviral therapy (ART) being central to reducing viral load which improves health, reduces transmission, and enhances life expectancy among people living with HIV (PLWH) [1–3]. According to the Joint United Nations Programme on HIV/AIDS (UNAIDS) in 2022, 86% of PLWH knew their status, 89% of those were receiving treatment, and 93% of those on treatment were virally suppressed, underlining progress but still falling short of the 95-95-95 targets [4, 5]. Achieving HIV virologic suppression requires considerable effort as it is contingent on performance at each stage of the HIV care continuum, especially on the initiation of ART and maintaining adherence to the treatment [6]. Self-efficacy, shaped by one’s experiences of control or lack thereof within one’s environment, is also recognized as a crucial factor in predicting adherence to ART [7–9]. Self-efficacy is an individual’s belief in their capacity to perform behaviors necessary to produce specific outcomes [9]. At the individual level, adherence to ART is affected by multiple factors, including psychological readiness, the particular ART regimen, the patient-provider relationship, social support, and access to services that enhance ART adherence [10–12]. However, less is known about the potential impact of one’s immediate physical and social environmental context—particularly perceived neighborhood disorder.

The socioecological theory (SET) posits that health is impacted by one’s environment [13]. The broken windows theory (BWT), under the umbrella of the SET, also suggests that minor signs of disorder and neglect, if unaddressed, create an environment where more disorder feels permissible [14]. This perception in turn impacts an individual’s health and health behaviors [15]. Neighborhood disorder is broken down into three categories, physical disorder, physical decay, and social disorder. Physical disorder refers to landscapes that contain trash, dirt on the street, abandoned cars, graffiti, vandalism, etc. while physical decay refers to structural characteristics such as abandoned buildings and deteriorated facilities [16]. Social disorder refers to events in public places seen as potentially threatening, such as fights, noise, homelessness, public drunkards, high levels of police activity, street prostitution, loitering individuals, and protests [16]. Collectively, neighborhood disorder is described as—observed or perceived physical and social features of neighborhoods that may signal the breakdown of order, and social control, and undermine the quality of life [16]. Studies have reported that constant exposure to stress related to living in a neighborhood where threat, crime, misconduct, trouble, and incivility are regular phenomena, may damage health and impact health behavior through psychological and physiological responses to stress [17, 18]. In the same vein, the place attachment theory suggests that the emotional bond people have with their neighborhoods can also affect their mental and physical well-being [19, 20].

Studies on the association between neighborhood characteristics and aspects of the HIV care continuum (diagnosis, linkage to care, ART initiation, retention in care, ART adherence, and HIV viral suppression) typically focus on objective measures such as neighborhood-level socioeconomic status (SES) [21, 22], deprivation [23, 24], poverty rates [25, 26], rural/urban status [27–29], crime rates [30, 31], incarceration rates [32], etc. which do not consider an individual’s experiences of their neighborhood [33]. Fewer studies have explored individual perceptions of neighborhoods and their relationship to the HIV care continuum [33]. Additionally, the perceived fear of crime has been linked to a reduced likelihood of having current ART prescriptions [34]. In this study, our objective was to assess the correlation between perceived neighborhood disorder, ART adherence self-efficacy, and HIV virologic suppression among people living with HIV. We hypothesize that a higher perception of neighborhood disorder would correlate with decreased ART adherence self-efficacy, and non-viral suppression.

## Methods

This was a cross-sectional survey of one hundred and eighty-eight individuals living with HIV who were on ART in Southeastern Arizona conducted from June 1, 2022 to February 6, 2023. Patients were recruited from two HIV clinics. Two recruitment strategies were employed. Patients were recruited through flyers at the reception area of the clinics and could self-administer the survey using a QR code. Alternatively, a member of the study team approached patients at the end of their clinic visit with the flyer with instructions on how to self-administer the one-time survey. Written informed consent, eligibility screening, and surveying were conducted electronically in that order. Study data were collected and managed using REDCap (Research Electronic Data Capture) [35, 36] hosted at the University of Arizona. After completion of the survey by eligible individuals, their most recent viral load result was abstracted from their medical records and combined with their survey data.

### Ethics statement

This study was approved by the Institutional Review Board (IRB) at the University of Arizona, Tucson (IRB number: STUDY00000098) on March 18, 2022. Each participant provided electronic written consent before participation.

### Eligibility criteria

The survey included PLWH aged 18 years of age or older, of all genders, who have been prescribed ART for at least 4 months [12], who had a viral load result within 12 months of study participation, at least four months after ART initiation [37], and who resided in Arizona for at least 12 months before the study. Participants who completed the survey received a $ 10 electronic gift card.

### Measures

We examined the relationship between perceived neighborhood disorder, ART adherence self-efficacy, and HIV virologic suppression, measured using validated scales.

Per*ceived neighborhood disorder* was measured using the validated Neighborhood Disorder Observation Scale [16, 38]. Neighborhood disorder in this scale was defined as conditions and activities that residents perceive to be cues or signs of the breakdown of social control with three sub-factors, physical disorder, physical decay, and social disorder. This is a 5-point scale where 0 is not present and 5 is highly present. The physical disorder factor of the scale (8 items) measures the physical appearance of a neighborhood. The physical decay factor (5 items) measures the condition of a neighborhood. The Social disorder factor (7 items) measures events in public places seen as potentially threatening creating a sense of danger [39]. For this study, we created three categories using average composite scores. No perception of disorder (Score of 0), Low perception of disorder (Score of >0–2.5), and finally high perception of disorder (Score of >2.5–5).

*ART adherence self-efficacy* was measured using the validated HIV Treatment Adherence Self-Efficacy Scale (HIV-ASES) [40]. This is a 12-item scale that assesses an individual’s confidence to carry out important treatment-related behaviors including adhering to treatment plans and medication regimen adherence in the face of barriers. The scale is rated on a 1–10 scale ranging; from 0 (cannot do it at all) to 10 (certain can do it) [40].

*Plasma HIV viral load data* were abstracted from the participant’s medical records. Virologic suppression was defined following the CDC guidelines that ≤200 RNA copies/ml constitutes virologic suppression [41]. Therefore, we categorized the viral load data per the recommendation for analysis.

Data on additional factors that may affect ART adherence were collected using validated scales, such as social support [42], alcohol and drug use [43], depression [44], HIV stigma [45], and provider-patient relationship [46]. Demographic data such as age, race/ethnicity, gender, education, household annual income, zip code, and length at the zip code were also obtained.

### Analysis

We applied the Fisher’s Exact Test to determine the association between two categorical variables. Continuous/ordinal variables were described using the median, interquartile range (IQR: 25th to 75th percentiles), and 95% confidence interval (95% CI). Measures of strength and direction of association between two ordinal variables were conducted using Spearman’s rank test. Additionally, the Wilcoxon rank sum test was employed for comparing medians between two independent groups. To identify factors associated with ART adherence self-efficacy among patients, we utilized Firth logistic regression with stepwise backward elimination, which mitigates small-sample bias. The categorization of ART adherence self-efficacy scores was driven by our data for the Firth logistic regression. We categorized high self-efficacy as scores for the firth logistic regression as: scores between 9 and10 as high and > 9 as lower ART adherence self-efficacy. This yielded 73 in the lower adherence self-efficacy group and 115 in the higher adherence self-efficacy group, allowing for more predictors in the model. Due to the limited number of virally unsuppressed participants (N = 10) in our sample, we emphasized ART adherence self-efficacy, which had greater variation and is a recognized factor of viral suppression [7–9]. This emphasis allowed meeting our research objective despite the sample size constraints. These methods were chosen based on the nature of our variables and sample size. Analyses were conducted using the STATA 17(49).

## Results

Among the 188 participants included in this analysis, most were middle-aged to older adults with a median age of 52 years. They were predominantly male (79%), white (62%), and identified as non-Hispanic (66%). Education levels among participants varied, with a substantial portion achieving at least a high school level education (85%). Forty one percent of participants were employed, while 38% were not in the workforce due to retirement or disability, and a smaller percentage (13%) were unemployed. Approximately half reported a household annual income of less than $25,000 annually (54%). The median of years participants had lived in their current zip code was 4 years.

Reported degrees of perceived neighborhood disorder varied significantly by several demographic variables and psychosocial variables (**Table 1**). Age (p<0.0001), education (p = 0.015), employment status (p = 0.048), household annual income (p<0.001), and length at the current zip code (p = 0.0016) significantly differed by the degree of perceived neighborhood disorder. Individuals with high perceived neighborhood disorder reported lower social support (median: 9, IQR: 6–10, p = 0.0065), higher depression scores (median: 16, IQR: 10–19, p = 0.0002), and higher HIV stigma scores (median: 3.1, IQR: 2.5–3.6, p = 0.0006). No significant differences in perceived neighborhood disorder were observed for gender, race, ethnicity, and patient-provider relationship. Notably, 7 of the 10 individuals who were virologically unsuppressed reported perceiving high disorder in their neighborhoods.

**Table 1:** Demographic and psychosocial characteristics by degree of perceived neighborhood disorder.

	No perceived disorder (Score 0)	Low perceived disorder (Score >0 and <2.5)	High perceived disorder (Score >2.5 and ≤ 5)	Total n (%) per group and median (IQR)	Spearman’s rho	p value	95% CI
Perceived neighborhood disorder scores^b^	n = 21 (11%)	n = 116 (62%)	n = 51 (27%)	188 (100%)			
Age (median (1QR))	62 (56–67)	53 (40–61)	44 (32–55)	52 (37–61)	-0.34	**<0.0001** *	(-0.4693 to -0.2203)
Gender^a^						0.843	
Female	2 (10%)	20 (17%)	8 (16%)	30 (16%)			
Male	19 (90%)	90 (78%)	40 (78%)	149 (79%)			
Other	0 (0%)	6 (5%)	3 (6%)	9 (5%)			
Race^a^						0.208	
White	17 (81%)	73 (63%)	27 (53%)	117 (62%)			
Non-White	4 (19%)	40 (34%)	21 (41%)	65 (35%)			
Prefer not to answer	0 (0%)	3 (3%)	3 (6%)	6 (3%)			
Ethnicity^a^						0.434	
Non-Hispanic	16 (74%)	80 (67%)	29 (57%)	125 (66%)			
Hispanic	5 (26%)	33 (28%)	21 (41%)	59 (31%)			
Prefer not to answer	0 (0%)	3 (3%)	1 (2%)	4 (2%)			
Education^a^						**0.015** *	
Less than high school	3 (14%)	6 (5%)	10 (20%)	19 (10%)			
High school	4 (19%)	51 (44%)	23 (45%)	78 (41%)			
More than high school	13 (62%)	52 (45%)	17 (33%)	82 (44%)			
Prefer not to answer	1 (5%)	7 (6%)	1 (2%)	9 (5%)			
Employment^a^						**0.048** *	
Unemployed	0 (0%)	16 (14%)	10 (20%)	26 (14%)			
Employed	7 (33%)	48 (41%)	22 (43%)	77 (41%)			
Other–Retired/disability	13 (62%)	46 (40%)	13 (25%)	72 (38%)			
Prefer not to answer	1 (5%)	6 (5%)	6 (12%)	13 (7%)			
Household annual income^a^						**<0.001** *	
< $25,000	4 (19%)	59 (50%)	38 (75%)	101 (54%)			
$25–50,000	8 (38%)	28 (24%)	8 (16%)	44 (23%)			
>$51,000	7 (33%)	22 (19%)	2 (4%)	31 (17%)			
Prefer not to answer	2 (10%)	7 (6%)	3 (6%)	12 (6%)			
Length of stay at zip code (median (IQR))	6 (3–12)	4 (2–15)	3 (1–6)	4 (2–10.5)	-0.22	**0.0016** *	(-0.3566 to -0.1016)
Frequency of tobacco use (median (IQR))	0 (0–0)	0 (0–3)	3 (0–4)	4 (0–4)	0.36	**<0.0001** *	(0.2462 to 0.4872)
Frequency of drugs use (marijuana, cocaine or crack, heroin, methamphetamine (crystal meth), hallucinogens, ecstasy/MDMA) (median (IQR))	0 (0–0)	0 (0–2)	2 (0–4)	0 (0–2.5)	0.25	**0.0003** *	(0.1190 to 0.4006)
Frequency of prescription drugs (median (IQR))	0 (0–0)	0 (0–0)	0 (0–1)	0 (0–0)	0.29	**<0.0001** *	(0.1421 to 0.4489)
Frequency of alcohol male use (median (IQR))	0 (0–0)	0 (0–1)	0.5 (0–1)	0 (0–1)	0.21	**0.0091** *	(0.0613 to 0.3659)
Frequency of alcohol female (median (IQR))	0 (0–0)	0 (0–1)	1 (0–1)	0 (0–1)	0.25	0.1743	(-0.0714 to 0.5828)
Viral load						**<0.0001** *	
Suppressed (<200 RNA copies/ml)	21 (100%)	110 (95%)	36 (71%)	167 (89%)			
Unsuppressed (>200 RNA copies/ml)	0 (0%)	3 (3%)	7 (14%)	10 (5%)			
Missing viral load	0 (0%)	3 (3%)	8 (16%)	11 (6%)			
Social Support (median (IQR))	10 (9–12)	10 (7–11)	9 (6–10)	9 (7–11)	-0.19	**0.0065** *	(-0.3317 to -0.0643)
Depression (median (IQR))	6 (3–9)	9.5 (4.5–16.5)	16 (10–19)	11 (5–17)	0.27	**0.0002** *	(0.1363 to 0.4055)
HIV stigma (median (IQR))	2.3 (1.2–3.0)	2.9 (2.2–3.4)	3.1 (2.5–3.6)	2.9 (2.2–3.4)	0.24	**0.0006** *	(0.1051 to 0.3895)
Personalized stigma (median (IQR))	1 (1–3)	2 (1–3)	2.6 (1.6–3.6)	2.3(1–3)	0.28	**0.0001** * *	(0.1485 to 0.4167)
Disclosure concerns (median (IQR))	3 (1.6–3.6)	3.6 (3–4.3)	3.3 (2.6–4)	3.6 (2.6–4.3)	0.03	0.6685	(-0.1200 to 0.1829)
Concerns about public attitudes (median (IQR))	2.6 (1–3.3)	3.3 (3–4)	3.6 (3–4.3)	3.3 (3–4)	0.26	**0.0002** *	(0.1297 to 0.4096)
Negative self-image (median (IQR))	1.6 (1–2.6)	2.3 (1.6–3.3)	3 (2–3.6)	2.3 (1.6–3.6)	0.22	**0.0020** *	(0.0744 to 0.3749)
Provider-patient relationship (median (IQR))	5 (4–5)	4.8 (4–5)	4.8 (4–5)	4.8 (4–5)	-0.00	0.9688	(-0.1491 to 0.1435)

Note

*Significant at p<0.05

^a^The percentages correspond to the column total and variable (*e*.*g*. *for the no perceived disorder group 10% were female and 90% were male*)

^b^The percentages correspond to the total number of participants (*e*.*g*. *for the no perceived disorder group*, *there were 21 (11%) of the total 188 participants*)

**Table 2** illustrates the relationship between HIV medication adherence self-efficacy and perceived neighborhood disorder. For those with no perceived disorder, the median score for integration of HIV medication into their routine was 10 with an IQR of 9.4–10, while for those perceiving high perceived disorder, it dropped to 8.4 with an IQR of 5.1–9.8. Similarly, for perseverance with HIV medication despite challenges, individuals with no perceived disorder had a median score of 10 with the same IQR, and this decreased to 8.3 (IQR: 5–10) for those with high perceived disorder.

**Table 2:** HIV medication adherence self-efficacy by degree of perceived neighborhood disorder.

	No perceived disorder (Score 0)	Low perceived disorder (Score >0 and <2.5)	High perceived disorder (Score >2.5 and ≤ 5)		
Perceived neighborhood disorder score	**n = 21**	**n = 116**	**n = 51**		
	**Median (IQR)**	**Median (IQR)**	**Median (IQR)**	**Spearman’s rho**	**p value**
**Integration**	10 (9.4–10)	9.5 (8.5–10)	8.4 (5.1–9.8)	-0.29	**<0.0001** *
Sticking to your treatment plan even when side effects begin to interfere with daily activities?	10 (10–10)	10 (8–10)	9 (5–10)	-0.26	**0.0002** *
Integrating your treatment into your daily routine?	10 (10–10)	10 (9–10)	9 (5–10)	-0.29	**<0.0001** *
Integrating your treatment into your daily routine even if it means taking medication or doing other things in front of people who don’t know you are HIV infected?	10 (10–10)	10 (7.5–10)	9 (5–10)	-0.23	**0.0009** *
Sticking to your treatment schedule even when your daily routine is disrupted?	10 (8–10)	10 (8.5–10)	9 (5–10)	-0.16	**0.0202** *
Sticking to your treatment schedule when you aren’t feeling well?	10 (9–10)	10 (8–10)	8 (5–10)	-0.24	**0.0006** *
Sticking to your treatment schedule when it means changing your eating habits?	10 (10–10)	10 (9–10)	9 (5–10)	-0.29	**<0.0001** *
Continuing with your treatment even if doing so interferes with your daily activities?	10 (10–10)	10 (9–10)	9 (5–10)	-0.25	**0.0004** *
Continuing with your treatment even when getting to your clinic appointments is a major hassle?	10 (10–10)	10 (9–10)	9 (5–10)	-0.26	**0.0003** *
Continuing with your treatment even when people close to you tell you that they don’t think that it is doing any good?	10 (10–10)	10 (9–10)	9 (5–10)	-0.26	**0.0003** *
**Perseverance**	10 (10–10)	9.6 (7.8–10)	8.3 (5–10)	-0.23	**0.0010** *
Continuing with the treatment plan your physician prescribed even if your T-cells drop significantly in the next three months?	10 (10–10)	10 (8–10)	9 (5–10)	-0.22	**0.0017** *
Continuing with your treatment even when you are feeling discouraged about your health?	10 (10–10)	10 (8–10)	10 (6–10)	-0.21	**0.0031** *
Getting something positive out of your participation in treatment, even if the medication you are taking does not improve your health?	10 (10–10)	10 (7–10)	8 (5–10)	-0.22	**0.0021** *

Note

• *Significant at p<0.05

• Questions are from a validated scale “*HIV Treatment Adherence Self Efficacy Scale”* [40]

In a Firth logistic regression analysis with stepwise backward elimination, factors associated with ART adherence self-efficacy were identified (**Table 3**). In the analysis, two variables remained statistically significant in the final model. Perceived neighborhood disorder was inversely associated with ART adherence self-efficacy, where individuals perceiving higher disorder had reduced odds of reporting higher ART adherence self-efficacy (OR: 0.67, 95% CI: 0.533–0.855, p = 0.001). Conversely, the quality of the patient-provider relationship was positively correlated with ART adherence self-efficacy, with stronger relationships being associated with higher odds of higher ART adherence self-efficacy (OR: 2.23, 95% CI: 1.402–3.564, p = 0.001). Furthermore, changes in routine resulting in missing ART for 48 hours or longer were significantly related to a decrease in ART adherence self-efficacy (OR: 0.29, 95% CI: 0.135–0.623, p = 0.002). Other factors, including tobacco and drug use, social support, depression, and HIV stigma, did not show significant associations with ART adherence self-efficacy in the final model.

**Table 3:** Predictors of ART adherence self-efficacy using Firth logistic regression with stepwise backward elimination.

Description	Odds Ratio	P-value	95% CI	Odds Ratio	P-value	95% CI
	Full Model			Final model		
**Perceived Neighborhood Disorder**	0.77	0.092	(0.575–1.042)	0.67	**0.001** *	(0.533–0.855)
**Tobacco Use**						
Never	Reference	---	---			
Less than monthly	1.40	0.631	(0.352–5.587)			
Monthly	4.14	0.301	(0.280–61.159)			
Weekly	1.32	0.742	(0.250–6.967)			
Daily or almost daily	2.27	0.094	(0.868–5.965)			
**Drug Use**						
Never	Reference	---	---			
Less than monthly	3.95	0.055	(0.969–16.110)			
Monthly	0.74	0.649	(0.213–2.616)			
Weekly	0.40	0.183	(0.109–1.525)			
Daily or almost daily	1.19	0.755	(0.390–3.649)			
**Social Support**						
Strong social support	Reference	---	---			
Poor social support	1.48	0.480	(0.496–4.433)			
Moderate social support	2.20	0.135	(0.782–6.187)			
**Depression**	0.97	0.295	(0.920–1.025)			
**HIV Stigma**	0.79	0.334	(0.507–1.259)			
**Patient-Provider Relationship**	2.40	**0.002** *	(1.368–4.227)	2.23	**0.001** *	(1.402–3.564)
**Reasons for missing ART**						
ART Access Problems (No)	Reference	---	---			
Yes	0.98	0.976	(0.296–3.247)			
Cost of meds/Clinic Care (No)	Reference	---	---			
Yes	0.39	0.318	(0.065–2.423)			
Lack of Food (No)	Reference	---	---			
Yes	0.91	0.859	(0.327–2.536)			
Experienced Side-Effects (No)	Reference	---	---			
Yes	0.82	0.616	(0.379–1.775)			
Change in routine for >48 hours (No)	Reference	---	---			
Yes	0.31	**0.017** *	(0.120–0.811)	0.29	**0.002** *	(0.135–0.623)

Note

*Significant at p<0.05

Perceived neighborhood disorder was significantly related to virologic suppression **(Table 4**). Individuals with suppressed viral loads (N = 167) reported significantly lower levels of disorder across multiple domains compared to those not suppressed (N = 10). Specifically, significant differences were observed in overall perceived neighborhood disorder (0.9 [IQR: 0.2–2.0] vs. 3.2 [IQR: 2.4–4], p = 0.0012), physical disorder elements such as graffiti (1 [IQR: 0–3] vs. 3 [IQR: 3–5], p = 0.0051), and trash in the street (2 [IQR: 0–4] vs. 4.5 [IQR: 3–5], p = 0.0255). Social disorder indicators like public intoxication (0 [IQR: 0–2] vs. 3 [IQR: 2–5], p = 0.0038) and drug selling (0 [IQR: 0–2] vs. 4 [IQR: 3–5], p = 0.0001) were also significantly elevated in the not suppressed group. Furthermore, indicators of physical decay such as vacant houses (0 [IQR: 0–2] vs. 3 [IQR: 2–5], p = 0.0010) and deteriorated recreation places (0 [IQR: 0–1] vs. 3 [IQR: 0–5], p = 0.0030) were significantly more prevalent in those without virologic suppression.

**Table 4:** Perceived neighborhood disorder by HIV virologic suppression (<200 RNA copies/ml).

	Suppressed (n = 167)	Not suppressed (n = 10)	
	Median (IQR)	Median (IQR)	p-value
**Perceived neighborhood disorder**	0.9 (0.2–2.0)	3.2 (2.4–4)	**0.0015** *
** Physical Disorder**	1.4 (0.4–2.8)	3.6 (2.2–4)	**0.0073** *
Cigarettes in the street	1 (0–3)	4 (2–4)	0.0564
Trash in the street	2 (0–4)	4.5 (3–5)	**0.0262** *
Empty bottles or cans in the street	2 (0–3)	5 (2–5)	**0.0193** *
Graffiti	1 (0–3)	3 (3–5)	**0.0062** *
Political or protest message graffiti	0 (0–2)	4.5 (3–5)	0.0772
** Social Disorder**	0.6 (0–2)	3.2 (2.8–3.6)	**0.0013** *
Adults or young people loitering	1 (0–3)	4.5 (3–5)	**0.0067** *
People drinking alcohol in public	0 (0–2)	2.5 (0–5)	0.1498
Gangs	0 (0–1)	2.5 (1–5)	**0.0006** * *
Public intoxication	0 (0–2)	3 (2–5)	0.0047*
Adults or young people fighting or arguing	1 (0–2)	3 (2–5)	**0.0008** *
The selling drugs	0 (0–2)	4 (3–5)	**0.0001** *
** Physical Decay**	0.2 (0–1.5)	2.8 (1.2–4.5)	**0.0028** *
Vacant houses	0 (0–2)	3 (2–5)	**0.0013** *
Abandoned, vandalized and run-down buildings	0 (0–2)	2 (1–5)	**0.0104** *
Deteriorated residential units	0 (0–2)	3 (0–5)	**0.0198** *
Deteriorated recreation places	0 (0–1)	3 (0–5)	**0.0039** *

**Note:** *Significant at p<0.05

Questions from a validated scale “*Neighborhood Disorder Observation Scale*” [16]

## Discussion

Achieving and sustaining HIV viral suppression requires adherence to the ART regimen, engagement in care to monitor viral load, and overcoming any barriers to these processes. Our study found that PLWH who perceive higher neighborhood disorder experience greater challenges with ART adherence self-efficacy than those who perceive less neighborhood disorder. Furthermore, we found that those who had not achieved virologic suppression reported higher perceptions of neighborhood disorder than those with virologic suppression. These findings affirmed the hypothesis and contribute to the growing evidence emphasizing the impact of community-level factors on health behavior and health outcomes [13] per the socio-ecological model.

The neighborhood disorder that individuals perceive speaks to elements of socioeconomic investments in those places. Health disadvantage goes beyond healthcare and can be attributed to policies, investments in neighborhoods, living conditions, and personal behaviors [47]. Therefore, those who perceived disorder in their neighborhoods saw less investment in aesthetics (e.g. deteriorated and abandoned buildings), public services (e.g street cleaning), and restoring social order (crime). In our study, those who perceived higher neighborhood disorder were younger, less employed, earning less, and had shorter stays in the current neighborhood compared to those who had lower perceptions of neighborhood disorder. Individual and neighborhood socioeconomic status has been associated with adherence self-efficacy, which in turn was related to ART adherence [48]. Additionally, high proportions of neighborhood unemployment and public assistance are associated with low levels of self-efficacy [49]. Thus, our findings are consistent with others that a higher perception of neighborhood disorder poses challenges to ART self-efficacy adherence. Our findings also show most of the individuals not virologically suppressed reported perceiving high neighborhood disorder which is consistent with the literature on neighborhood-level factors associated with HIV viral suppression status [50–52]. HIV viral suppression is reliant on ART adherence, and adherence is reliant on sustained HIV care engagement [53]. Neighborhood quality perceptions have been linked to utilization of care, as a study found that individuals with more unfavorable perceptions of their physical environment were significantly more likely to report a lack of a usual source of care and longer periods since the last routine visit [54]. Disorderly neighborhoods may also lack proper infrastructure, such as well-maintained roads and public transportation options. This can create transportation challenges for residents when accessing healthcare facilities.

A major strength of our study lies in its utilization of validated measures. Additionally, our study’s focus on the underexplored intersection of these factors fills a critical knowledge gap, thereby contributing to the broader understanding of HIV prevention and treatment. Despite these strengths, limitations are present. First, this is a cross-sectional study; therefore, causality cannot be established. Second, the sample size for those not virologically suppressed was relatively small, potentially limiting the scope of the statistical analysis and the generalizability of these results. Since our sample had only 10 virally unsuppressed participants in our sample, we chose to emphasize ART adherence self-efficacy, which exhibited more variation and is a known determinant of achieving viral suppression [7–9]. This approach provided valuable insights into how environmental factors impact ART adherence confidence, which is essential for viral suppression. Focusing on ART adherence self-efficacy, we aimed to highlight a key pathway through which neighborhood disorder affects HIV treatment outcomes, addressing the research objective within the constraints of our sample size. Lastly, responses to the survey instruments were self-reported, which may be subject to individual biases.

Our study highlights the influence of community-level factors such as perceived neighborhood disorder on health behavior and outcomes among PLWH in line with the SET. Understanding that higher levels of perceived neighborhood disorder have a bearing on ART adherence self-efficacy needed for achieving HIV viral suppression provides an opportunity for public health interventions to focus on improving neighborhood conditions as a strategy for promoting health. As neighborhood disorder is often a manifestation of underinvestment in community resources, policy interventions may focus on increasing investment in affected neighborhoods. With neighborhood quality closely tied to healthcare engagement, efforts should also be made to improve the safety and quality of public spaces and services to facilitate better access to healthcare facilities. Our study’s findings point to a complex interplay of social, environmental, and individual factors affecting ART adherence and viral suppression. This calls for a multifaceted public health strategy incorporating both macro- and micro-level interventions aimed at improving not only healthcare outcomes but also the broader social determinants of health.

## Conclusion

The challenges in HIV management are not limited to the individual but extend to the community and society at large. Policymakers and public health officials must recognize the role of neighborhood conditions in health outcomes and develop strategies that address these external factors by doing so, we can offer a more holistic and effective approach to managing HIV and improving the lives of those affected.

## Supporting information

S1 Table Predictors of ART adherence self-efficacy using Firth logistic regression with stepwise backward elimination. (DOCX)
