# Supplementary material for: Perceived neighborhood disorder and achieving HIV viral suppression among adults living with HIV: A cross-sectional study
Source: PLOS Glob Public Health. 2024 Dec 19;4(12):e0004060. doi: 10.1371/journal.pgph.0004060 (PMC11658497; doi:10.1371/journal.pgph.0004060)
Supplement: S1 Table — (DOCX) [file pgph.0004060.s001.docx]

| **S1 Table: Predictors of ART adherence self-efficacy using Firth logistic regression with stepwise backward elimination.** | | | | | | |
| --- | --- | --- | --- | --- | --- | --- |
| **Description** | **Odds Ratio** | **P-value** | **95% CI** | **Odds Ratio** | **P-value** | **95% CI** |
|  | **Full Model** | | | **Final model** | | |
| **Perceived Neighborhood Disorder** |  |  |  |  |  |  |
| No perceived disorder | Reference | --- | ---- |  |  |  |
| Low perceived disorder | 0.33 | 0.096 | (0.090 - 1.215) | 0.42 | 0.162 | (0.125 - 1.415) |
| High perceived disorder | 0.22 | 0.054 | (0.047 - 1.024) | 0.17 | **0.009*** | (0.048 - 0.644) |
| **Tobacco Use** |  |  |  |  |  |  |
| Never | Reference | --- | --- |  |  |  |
| Less than monthly | 1.53 | 0.551 | (0.374 - 6.303) |  |  |  |
| Monthly | 2.84 | 0.448 | (0.191 - 42.341) |  |  |  |
| Weekly | 1.22 | 0.812 | (0.231 - 6.469) |  |  |  |
| Daily or almost daily | 2.41 | 0.073 | (0.920 - 6.320) |  |  |  |
| **Drug Use** |  |  |  |  |  |  |
| Never | Reference | --- | --- |  |  |  |
| Less than monthly | 4.51 | **0.040*** | (1.073 - 18.989) |  |  |  |
| Monthly | 0.85 | 0.808 | (0.241 - 3.028) |  |  |  |
| Weekly | 0.36 | 0.140 | (0.096 - 1.389) |  |  |  |
| Daily or almost daily | 1.08 | 0.883 | (0.362 - 3.253) |  |  |  |
| **Social Support** |  |  |  |  |  |  |
| Strong social support | Reference | --- | --- |  |  |  |
| Poor social support | 1.53 | 0.446 | (0.510 - 4.607) |  |  |  |
| Moderate social support | 2.38 | 0.107 | (0.829 - 6.860) |  |  |  |
| **Depression** | 0.97 | 0.285 | (0.920 - 1.024) |  |  |  |
| **HIV Stigma** | 0.82 | 0.404 | (0.514 - 1.307) |  |  |  |
| **Patient-Provider Relationship** | 2.63 | **0.001*** | (1.456 - 4.758) | 2.25 | **0.001*** | (1.412 - 3.610) |
|  |  |  |  |  |  |  |
| **Reasons for missing ART** |  |  |  |  |  |  |
| ART Access Problems (No) | Reference | --- | --- |  |  |  |
| Yes | 0.96 | 0.952 | (0.287 - 3.230) |  |  |  |
| Cost of meds/Clinic Care (No) | Reference | --- | --- |  |  |  |
| Yes | 0.41 | 0.353 | (0.065 - 2.646) |  |  |  |
| Lack of Food (No) | Reference | --- | --- |  |  |  |
| Yes | 0.84 | 0.747 | (0.308 - 2.327) |  |  |  |
| Experienced Side-Effects (No) | Reference | --- | --- |  |  |  |
| Yes | 0.89 | 0.774 | (0.412 - 1.935) |  |  |  |
| Change in routine for >48 hours (No) | Reference | --- | --- |  |  |  |
| Yes | 0.29 | **0.013*** | (0.112 - 0.773) | 0.26 | **0.001*** | (0.124 - 0.570) |
| **Note:** *Significant at p<0.05 | | | | | | |

In **Table 3a**, the final model identified key predictors of ART adherence self-efficacy using Firth logistic regression with stepwise backward elimination. High levels of perceived neighborhood disorder were associated with significantly lower odds of ART adherence self-efficacy (OR = 0.17, 95% CI: 0.048–0.644, p = 0.009), suggesting that individuals who perceive their neighborhoods as highly disordered may have reduced confidence in adhering to ART regimens. Although low perceived neighborhood disorder (OR = 0.42, p = 0.162) indicated a similar trend, it was not statistically significant. A strong patient-provider relationship emerged as a positive predictor, with those experiencing high support from their healthcare providers being more likely to report confidence in ART adherence (OR = 2.25, 95% CI: 1.412–3.610, p = 0.001). Additionally, individuals who faced disruptions in their routine impacting ART adherence had lower odds of ART adherence self-efficacy (OR = 0.26, 95% CI: 0.124–0.570, p = 0.001), underscoring the negative impact of routine instability. Tobacco and drug use, social support, depression, and other ART-related challenges were not retained in the final model.
